# From nucleotides to intact transcripts through oligonucleotides: Integrated chromatographic strategies for therapeutic mRNA characterization

**DOI:** 10.1007/s00216-026-06615-x

**Published:** 2026-06-23

**Authors:** Mar Losada, Gemma Gotor, Salvador Borrós, Cristina Fornaguera

**Affiliations:** Grup d’Enginyeria de Materials (Gemat), Institut Químic de Sarrià (IQS), Universitat Ramon Llull (URL), Barcelona, Spain

**Keywords:** mRNA-based therapeutics, Liquid chromatography-mass spectrometry (LC-MS), Critical quality attributes (CQAs), Oligonucleotide mapping, mRNA characterization, Analytical workflows

## Abstract

Messenger RNA (mRNA) has emerged as a transformative modality for modern therapeutics, including vaccines and gene therapies. Ensuring the quality, safety, and efficacy of mRNA products requires the evaluation of critical quality attributes (CQAs) such as sequence integrity, nucleotide modifications, purity, and structural stability. The growing complexity of modified mRNA therapeutics highlights the need for robust and orthogonal analytical strategies. This review summarizes liquid chromatography (LC)-based workflows tailored for mRNA characterization including UV detection and mass spectrometry (MS) strategies. These include nucleoside-level analysis to identify chemical modifications and impurities, partial digestion with oligonucleotide mapping to assess sequence coverage and localization of modifications, and intact mRNA analysis to evaluate global structural integrity and encapsulation efficiency. Case studies illustrate practical applications by directly linking analytical workflows to specific CQAs and regulatory-relevant attributes. By leveraging advances in chromatography and mass spectrometry—including bioinert stationary phases, high-resolution detection, and ion-pairing strategies—these studies enable robust analysis of highly polar, charged, and high-molecular-weight mRNA species. The integration of complementary chromatographic and MS-based workflows supports comprehensive characterization, facilitates regulatory compliance, and informs process and formulation development, ultimately providing a coherent framework for quality control in emerging mRNA therapeutics.

**Graphical abstract:**

**Figure:**
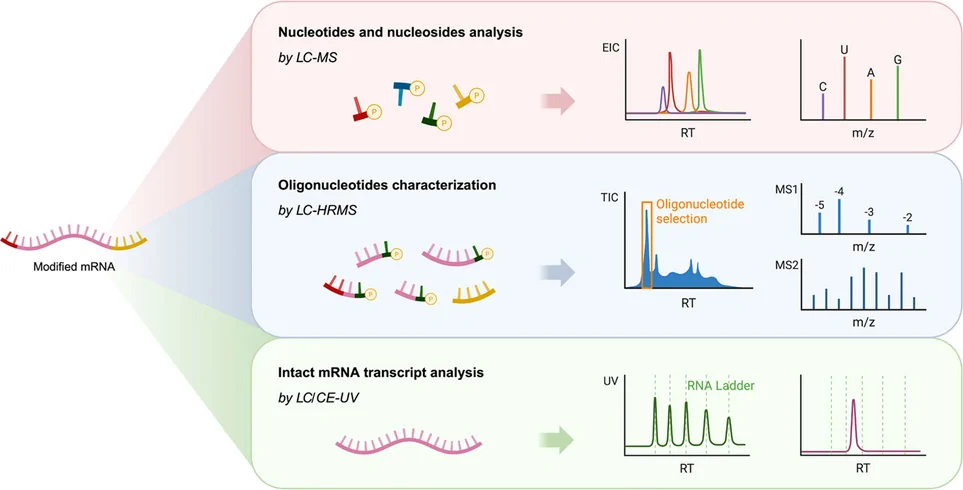

**Supplementary Information:**

The online version contains supplementary material available at https://doi.org/10.1007/s00216-026-06615-x.

## mRNA therapeutics: state of the art

Messenger RNA (mRNA)-based therapies have emerged as a platform that has transformed modern medicine in recent decades. Claimed as promising at the beginning of the 2000s, their efficacy was not proven until the onset of COVID-19, where they enabled massive vaccination in a record time development stage. Thus, although they gained prominence during the pandemic and became known for their rapid approval, they have been under development for more than two decades, primarily focusing on cancer treatment and immunotherapy, as well as for gene therapy approximations [1–4]. These novel therapies are emerging as alternatives to long-established and widely used small-molecule-based treatments. The development of low-molecular-weight chemical compounds and the search for the perfect molecule has constituted the basis of pharmacology. Although these compounds can cross cellular membranes and act on intracellular targets, they often exhibit low specificity, are prone to resistance, and can require chronic administration to achieve therapeutic efficacy. This prolonged use can result in systemic effects and cumulative toxicity, which may lead to undesirable adverse effects [5].

mRNA-based therapies allow the host cells to produce the therapeutic agent directly. Once inside the cytoplasm, the mRNA is translated into the encoded protein, meaning that simple delivery of a nucleotide sequence is sufficient to drive in situ production of the protein of interest. This represents one of the major advantages of mRNA systems: the sequence can be easily engineered and customized to express virtually any desired protein in the target cell. Such proteins may be endogenous factors required for physiological correction, tumor-associated antigens, and monoclonal antibodies or their fragments, as well as cytokines, enzymes, or receptors. Furthermore, mRNA does not integrate into the genome, as is naturally degraded. This high degree of versatility and capacity for customization is particularly valuable in cancer immunotherapy, where the intrinsic heterogeneity of tumor cells requires tackling several aberrant mutations at once [6].

Despite these clear advantages, the main limitations of mRNA were already detected in the early studies in the 1990s, which first demonstrated that exogenously administered mRNA could drive in vivo protein expression [7]. The major challenges include its intrinsically low chemical and structural stability, strong innate immunogenicity, and inefficient intracellular delivery, due to the poor capacity of naked mRNA to cross cellular membranes. Since then, extensive research efforts have been focusing on overcoming these limitations through two complementary strategies: (i) encapsulating mRNA within nano-delivery systems to protect it and facilitate cellular uptake, and/or (ii) chemically or structurally modifying the mRNA molecule itself to enhance stability and reduce immunogenicity [3, 8–11]. In the present review, we focus on this second approach.

## Modifying the mRNA to improve its stability

**Fig. 1:**
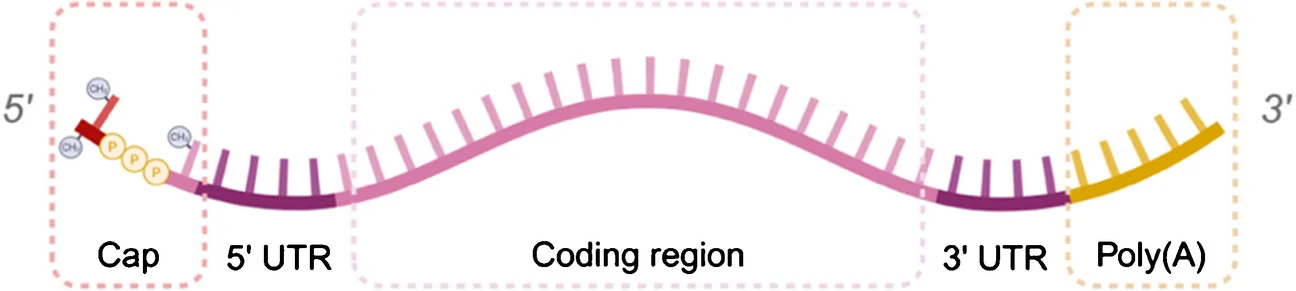
Structural elements of mRNA, including 5′ cap, untranslated regions (UTRs), and 3′ poly(A)

One of the first key breakthroughs during the 2000s was the identification that nucleoside modifications can reduce innate immune activation. Among the most relevant modifications in nucleobases is pseudouridine (Ψ), which decreases activation of the major mediators of immune response and enhances translational efficiency [12]; N1-methyl-pseudouridine (m^1^Ψ), which improves the protein production and increases in vivo tolerability [13]; and 5-methylcytosine (m^5^C), which not only reduces immune recognition but also improves transcript conformation [14]. Another critical feature is the presence of a modification at the 5′ end of the transcript. Viral mRNAs naturally exhibit a cap 0 structure (m^7^GpppN), consisting of a reversed methyl-guanosine linked by a triphosphate bridge. In eukaryotes, this structure is further modified into cap 1, which includes a methylation in the ribose of the first nucleotide, or cap 2, which adds an additional methylation at the second nucleotide. These cap structures are essential for protecting the mRNA from exonuclease-mediated degradation and for efficient ribosome recognition [15]. Untranslated regions (UTRs) are also present at both ends of the mRNA molecules, located at the 5′ and 3′ termini. These regions must be carefully optimized to ensure efficient translation initiation, to minimize the formation of secondary structures, to enhance cytoplasmic stability, and to increase the number of translational cycles [16]. At the 3′ end of the transcript, a consecutive stretch of adenosines, typically ranging between 100 and 150 nucleotides, forms the poly(A) tail. It can be entirely constructed of adenosines, or it may include short combinations of the other nucleotides, which can influence its structural properties and functional performance. This element plays a crucial role in protecting the mRNA from exonucleases and in promoting efficient translation [17]. They are all summarized in Fig. 1.

To address the challenges with the limited stability of mRNA and to introduce specific modifications that enhance transcript performance, protocols have been developed for the in vitro synthesis of chemically modified mRNA, commonly referred to as in vitro transcription (IVT). The process begins with the design of a DNA plasmid containing the regions of interest, including the nucleotide sequence encoding the target protein and elements that enable the incorporation of chemical modifications. The plasmid is then linearized using restriction enzymes to generate a linear double-stranded DNA template. This DNA is then purified and prepared for mRNA synthesis, where, through the action of RNA polymerases, usually T7, and the addition of nucleoside triphosphates, the DNA strand is transcribed into RNA. Following transcription, multiple purification steps are required to remove residual DNA template, unincorporated nucleotides, enzymes, and other reaction components, ensuring the production of high-quality mRNA suitable for downstream applications [18–20].

Some of the modifications, such as the 5′ cap or the 3′ poly(A) tail, can be added post-transcriptionally through enzymatic reactions. Nucleotide modifications, on the other hand, are incorporated directly during transcription by using pre-modified nucleoside triphosphates added to the mix. This co-transcriptional addition during the synthesis can also be used to incorporate the 5′ cap, where different capping analogs have been developed, allowing the direct addition of the modified molecule without requiring an enzymatic reaction and avoiding intermediate products [21]. In the case of the poly(A) tail, it can be included directly during the design of the DNA plasmid, where it is added consecutively following the 3′ UTR region.

The production of modified mRNA improves stability, increases the translation efficiency, and also reduces immunogenicity.

**Fig. 2:**
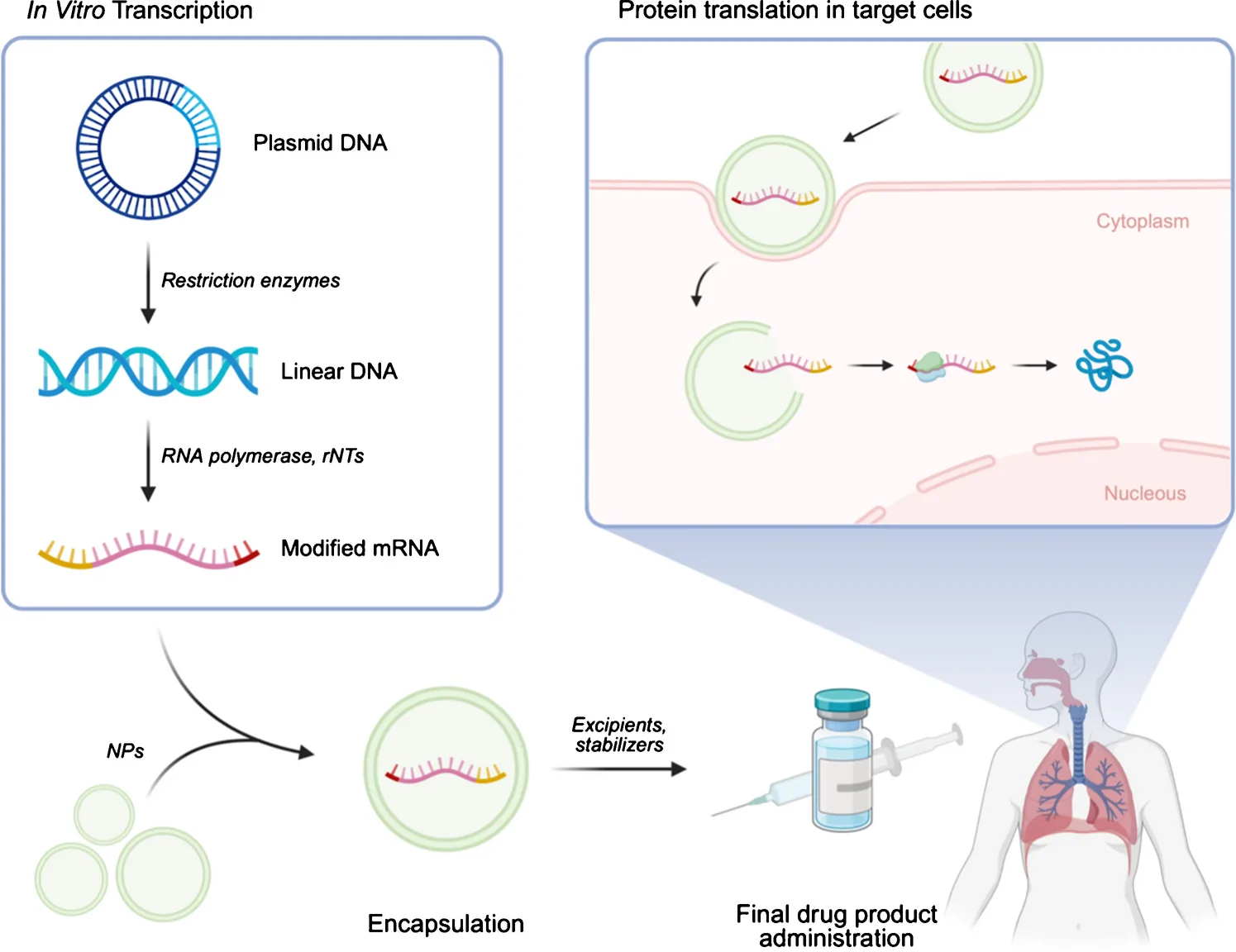
Overview of the mRNA vaccine workflow, including in vitro transcription (IVT), mRNA modifications, nanoparticle encapsulation, administration into the body, transport to target cells, cytoplasmic delivery, and subsequent protein expression

Figure 2 provides a schematic overview of the mRNA production and delivery process, from plasmid DNA and in vitro transcription, through incorporation of nucleotide modifications and encapsulation into nanoparticles, to administration, cellular uptake, cytoplasmic release, and translation into the protein of interest.

## Critical quality attributes (CQAs) of mRNA-based therapeutics

Given the structural and functional complexity of mRNA, regulatory agencies emphasize the need to systematically monitor critical quality attributes (CQAs) to ensure product safety and efficacy. Initially, regulatory authorities did not provide specific criteria for these biomolecules; instead, compliance had to be aligned with the existing general guidelines for biologics and vaccines, such as the International Conference on Harmonisation (ICH) Q6B [22]. The urgency to define and standardize quality requirements increased substantially in 2020 with the introduction of mRNA vaccines against COVID-19. The World Health Organization (WHO) published regulatory guidance in 2020 on the quality, safety, and efficacy of mRNA vaccines, including some definition of CQAs as part of the overall quality assessment [23]. In February 2022, the United States Pharmacopeia (USP) released the first draft of specific guidance [24]. This draft included generic analytical methods and a preliminary set of CQAs. These proposals were further expanded in the second and third drafts released in 2023 and 2024, which also introduced methodological criteria for defining CQAs for mRNA as a drug substance and as a drug product, defining the modified mRNA as the active pharmaceutical ingredient (API). Those include analytical approaches to characterize the complete nanosystem when the mRNA is delivered using a carrier [25, 26]. In parallel, the European Medicines Agency (EMA) published a concept paper in June 2023 to initiate the development of scientific guidelines addressing mRNA-specific quality considerations. This included manufacturing, characterization, and analytical control for vaccines [27]. This effort culminated in March 2025 with the publication of the first draft guideline on mRNA quality which formally defined CQAs as the attributes that must be monitored for release of the active substance [28]. In 2024, the European Pharmacopoeia (Ph. Eur.) approved the first general texts on mRNA vaccines, which included sections on quality and control defining quality attributes [29]. In July 2025, three general texts were published separately regarding mRNA-based therapeutics:

1. mRNA vaccines for human use (5.36), covering mRNA packaged in lipid nanoparticles
2. mRNA substances for the production of mRNA vaccines for human use (5.39), covering mRNA active substances used in the manufacture of mRNA vaccines
3. DNA templates for the preparation of mRNA substances (5.40), covering the linear DNA template used as starting material for the preparation of mRNA substances

They all cover the definition of the drug production, along with the required tests and assays for both mRNA vaccine as a drug product or the mRNA itself as a drug substance [30]. Ultimately, the aim is to integrate these studies and drafts, summarized in Table 1, into a harmonization set of CQA criteria for mRNA within functional ICH guidelines.

**Table 1:** Summary of relevant regulatory guidelines

Date	Organism	Document and Edition	Key notes	Ref.
October 2021	WHO	Evaluation of the quality, safety, and efficacy of mRNA vaccines (TRS 1039)	Global regulatory guidance	[23]
February 2022	USP	Analytical Procedures for mRNA Vaccine Quality – 1st draft edition	Proposal of initial analytical methods	[24]
April 2023	USP	Analytical Procedures for mRNA Vaccine Quality – 2nd draft edition	Expanded method-driven guides	[25]
June 2023	EMA	Concept paper on quality aspects of mRNA vaccines	Regulatory planning, only conceptual	[27]
August 2024	USP	Analytical Procedures for mRNA Vaccine Quality – 3rd draft edition	Most detailed analytical framework across lifecycle	[26]
November 2024	Ph. Eur.	Compendial standards	First compendial texts on mRNA vaccines approved	[29]
March 2025	EMA	Regulatory quality guideline	Attribute-driven regulatory focused	[28]
July 2025	Ph. Eur.	General chapters: 5.36 (Drug Product), 5.39 (Drug Substance), 5.40 (DNA template)	Legally binding compendial framework	[30]

Across these guidelines, a clear distinction has been made between CQAs for the mRNA drug substance and those for the drug product, which includes both the carrier and the mRNA, enabling assessment of the full nanosystem. This classification could—and should—also consider the diversity of nanosystems that may be employed for mRNA encapsulation.

**Fig. 3:**
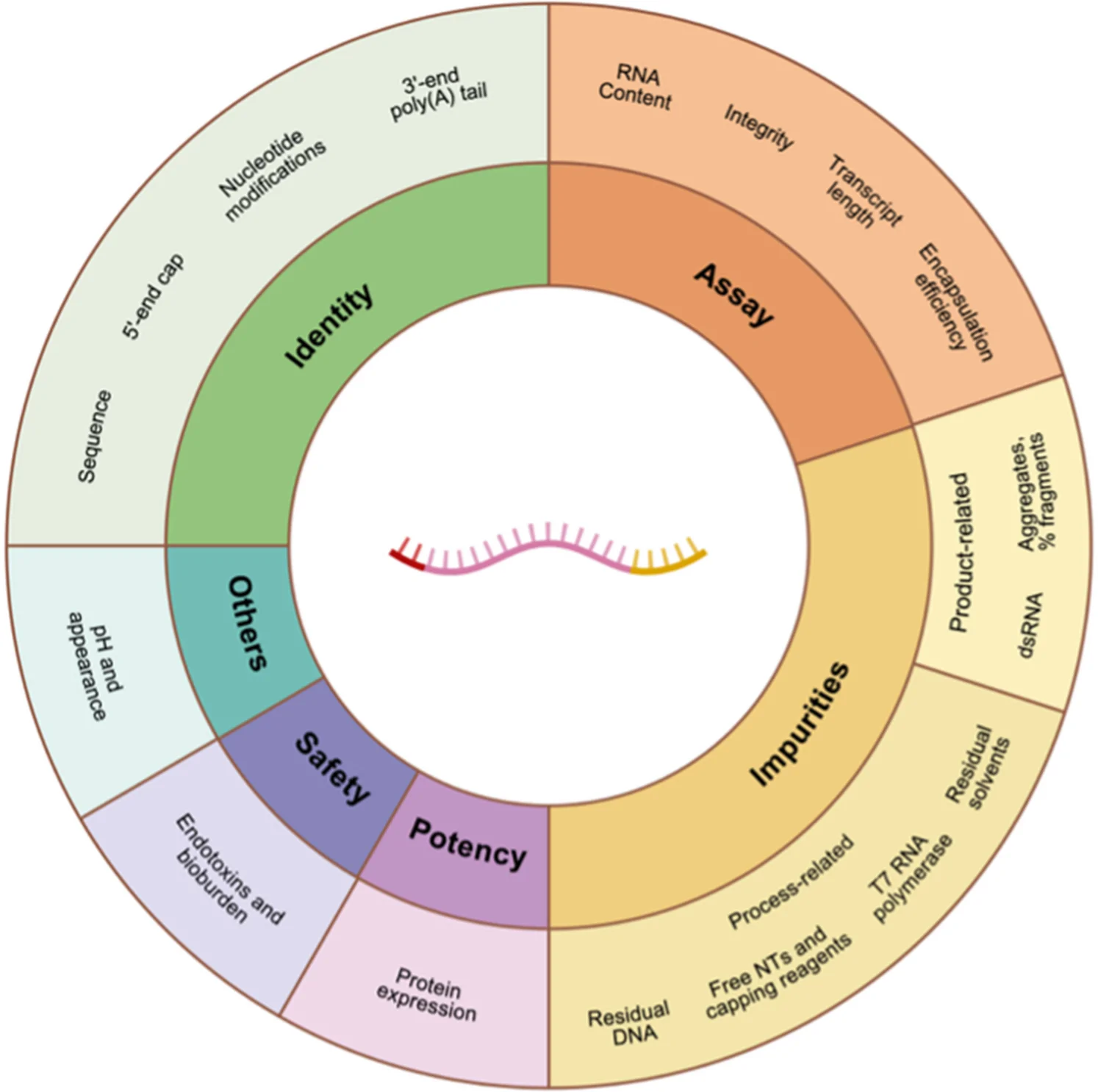
Critical quality attributes (CQAs) associated with the modified mRNA

By reorganizing the previously mentioned proposals around mRNA as the drug substance and classifying the relevant attributes, the resulting classification is presented in Fig. 3.

When considering CQAs related to the drug substance, several distinct aspects can be identified. (i) *Identity*, which includes the nucleotide sequence confirmation, the determination of the 5′ cap, characterization of nucleotide modifications, and assessment of the 3′ poly(A) tail length and presence. These chemical QCAs define the molecular subcomponents that constitute the mRNA and establish its identity. (ii) *Purity and integrity*, which involve determining mRNA concentration, intactness, stability, transcript integrity, and overall length. These attributes characterize the mRNA transcript as single unit, functional entity, and the active pharmaceutical ingredient. (iii) *Impurities*, which are categorized as product-related and process-related. *Product-related impurities* include the determination of dsRNA, aggregates, and the mRNA fragment percentages. These refer to sub-products that may be formed, often of the same nature as the active ingredient. In contrast, *process-related impurities* include residual DNA, non-incorporated nucleotides, residual capping agents, residual RNA polymerase remands, and residual solvents, among others. These refer to residual products that may remain after synthesis and purification. (iv) *Potency,* typically assessed through mRNA expression studies that monitor production of the encoded protein. (v) *Safety attributes*, which include endotoxin and bioburden levels to evaluate the potential hazard associated with the construct. (vi) *Physicochemical parameters* such as pH and appearance. While identity and purity primarily describe the mRNA as the analyte of interest, the behavior of this large biomolecule within the formulation matrix must also be considered when assessing all additional attributes.

## Analytical challenges in biomolecule characterization

The main analytical challenges arise from the intrinsic nature and the size of the analyte—the mRNA transcript. In vitro transcription (IVT) generates a single-stranded RNA molecule in which ribose sugars are linked through phosphate groups. The ribose contains a 2′-hydroxyl group that increases polarity related to DNA which lacks this functional group. This hydroxyl group can form additional hydrogen bonds with water or other phosphate groups. The phosphate backbone carries acidic protons that, at physiological pH, are largely deprotonated, conferring a net negative charge that further enhances the molecules hydrophilicity and polarity. This negative charge can be partially neutralized by cations such as Na^+^, Mg^2+^, or K^+^. Overall, these structural features render mRNA a highly polar and water-soluble biomolecule [31].

**Fig. 4:**
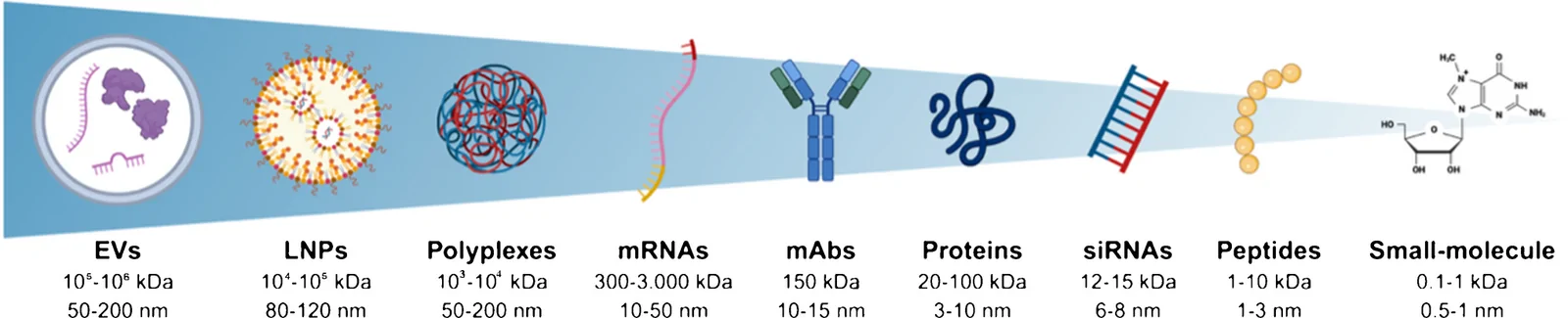
Comparative scale of representative therapeutic entities, from nanoparticles to small molecules, shown by approximate size (nm) and molecular weight (in kDa)

Typical mRNA transcripts range from approximately 1000 to 10,000 nucleotides in length, depending on the protein they encoded. Each amino acid corresponds to a codon of three nucleotides, not including the untranslated regions (UTRs) at the 5′ and 3′ ends, or the poly(A) tail. Within this size range, mRNA molecular weights span approximately between 300 kDa and 3000 kDa, classifying them as a large biomolecule, as illustrated in Fig. 4. Another important distinction between mRNA and other commonly characterized biomolecules—such as proteins—lies in their building blocks. While proteins have a combinatorial diversity of 21 amino acids (plus selenocysteine), mRNA is composed of only four nucleobases, a feature that has significant implications for bottom-up analytical strategies. The considerable size and structural complexity of mRNA are precisely the factors that have required adaptations of standard analytical techniques for small molecules, including liquid chromatography (LC) and mass spectrometry (MS), to enable their reliable characterization.

Since its inception in the 1960s–1970s, liquid chromatography was originally designed to retain small hydrophilic molecules (< 1 kDa). Early chromatographic columns featured small pore sizes (60–120 Å), silica-based stationary phases, and metallic surfaces prone to nonspecific adsorption, which limited compatibility with large biomolecules or highly charged analytes. During the 1980s, the field expanded with the introduction of diverse stationary phases with different mechanisms of retention—such as reversed-phase (RP), ion-exchange (IEX), and size-exclusion chromatography (SEC)—and the increase of pore sizes up to 300 Å. More robust stationary phases provided improved control over pH and ionic strength, enabling the retention and analysis of peptides and proteins. Nevertheless, strong nonspecific adsorption, interaction with metallic surfaces, and the use of denaturing conditions continued to limit the evaluation of certain analytes [32]. A major turning point occurred in the 1990s with the emergence of electrospray ionization (ESI) and matrix-assisted laser desorption/ionization (MALDI). Coupling chromatographic separations with mass spectrometry allowed the detailed characterization of intact proteins and peptides, as multiply charged ions enabled accurate molecular weight determination [32]. From the 2000s onwards, the rise of protein and antibody-based therapeutics drove the development of bioinert chromatographic systems to minimized adsorption, oxidation, and signal loss. Materials such as passivated steel, titanium, or polyether ether ketone (PEEK) were introduced. Alongside these new surfaces, stationary phases capable of retaining more polar molecules were developed using MS-compatible solvents, including hydrophilic interaction chromatography (HILIC), with different stationary phase ends. In parallel, ion-pairing reversed-phase chromatography (IP-RP) was adapted for charged compounds through the use of oppositely charged ion-pairing agents, including oligonucleotides, siRNA, and other nucleic acids [33]. From 2020 to the present, chromatographic development has undergone a significant inflection point, with the introduction of much larger pore sizes (> 1.000–2.000 Å) tailored for fragile and highly charged molecules [34]. Chromatography and mass spectrometry systems have been further optimized to ensure fully bioinert surfaces, improving robustness and reproducibility. Collectively, these technological advances have been successfully adapted to meet the complex challenges associated with mRNA and related analytes [35].

## LC-based analytical workflows for mRNA

**Fig. 5:**
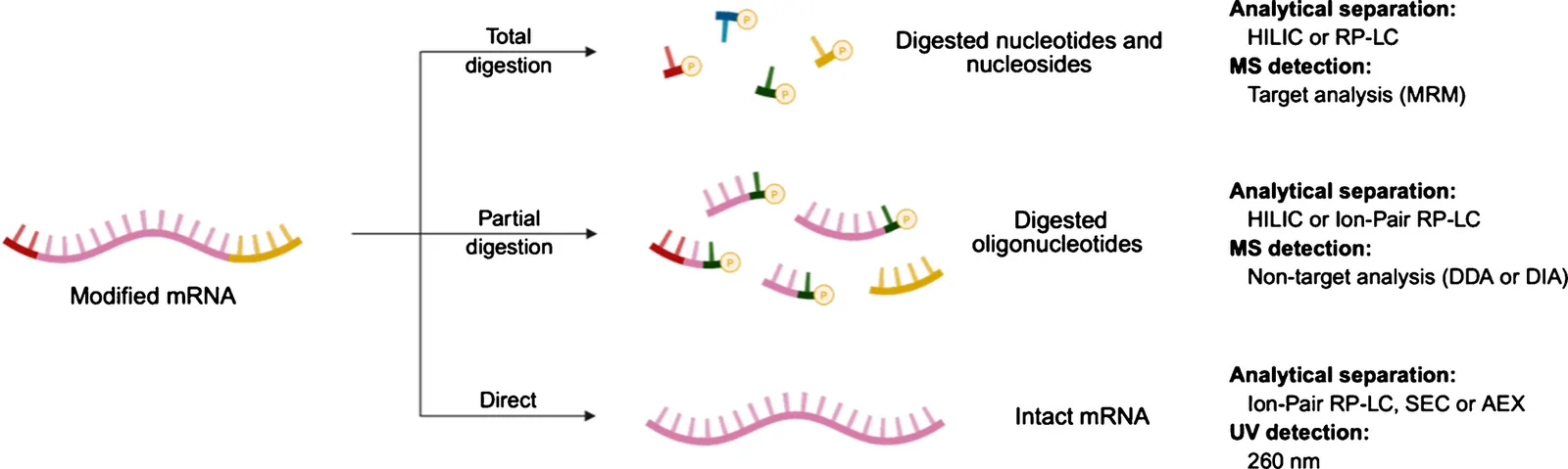
Analytical workflows used for mRNA characterization

To address the complexity of modified mRNA and its associated critical quality attributes, three complementary liquid chromatography methods with MS and UV detection workflows were established, as seen in Fig. 5, spanning different molecular levels of information. These workflows range from nucleoside-level analysis following total digestion, through oligonucleotide mapping after controlled partial digestion, to intact mRNA analysis for global integrity assessment. Together, they provide an orthogonal and comprehensive analytical framework for mRNA therapeutics, mainly covering the identity attributes.

### Workflow I: total digestion for nucleotide-level characterization

#### Sample preparation to obtain small-molecule-like chromatographic and MS-detectable analytes

Total digestion of mRNA can be performed with nonspecific ribonucleases such as nuclease P1. This zinc-dependent single-strand endonuclease hydrolyzes RNA phosphodiester bonds without base specificity, generating a mixture of ribonucleoside monophosphates. These monophosphates behave as small-molecule-like analytes, with molecular weights typically ranging from 320 to 370 Da and logP between −3.5 and −4.5. They are highly polar species that can carry a negative charge on the phosphate group depending on the medium pH.

This analytical group also includes species that may be present in the final product, such as nucleoside triphosphates—initial reagents of the IVT reaction—and nucleoside diphosphates, which can arise as degradation products. These compounds exhibit higher molecular weight ranges, approximately from 480 to 530 Da for triphosphates and from 400 to 450 Da for diphosphates, and logP around −6 for triphosphates and −4.5 for diphosphates. When further molecular simplification is required, particularly to remove the phosphate group responsible for the negative charge, phosphatases can be employed to convert nucleotides into nucleosides. Several phosphatases are available, differing in their extraction origin, such as shrimp alkaline phosphatase or calf intestinal alkaline phosphatase. Most of these enzymes efficiently remove phosphorylated termini from both DNA and RNA monophosphates, whether located at the 5′ or 3′ end [36]. This secondary enzymatic step yields a pool of nucleosides with moderate polarity with logP between −1 and −2, and molecular weights ranging between 240 and 290 Da. Alternatively, mRNA can be subjected to chemical hydrolysis under acidic conditions and elevated temperatures, which may induce cleavage of the *N*-glycosidic bond, yielding free nucleobases depending on the severity of the conditions [37, 38]. These nucleobases range between 110 and 150 Da and logP between 0 and −2. The polarity, exact mass, and chemical formula of the four canonical nucleotides as well as the most relevant nucleotide modifications are summarized in Supplementary Table S1 for each individual compound.

#### LC methods

For analytical separation, surface bio-inertization and the adaptation of chromatographic columns are critical when dealing with highly polar analytes carrying intrinsic charges. A clear distinction must be made between the separation of nucleotides, including mono-, di-, and triphosphates, as well as capping reagents, all containing phosphate groups, and the separation of nucleosides or nucleobases, which lack these functionalities. Consequently, these classes of compounds require distinct chromatographic strategies.

Hydrophilic interaction liquid chromatography (HILIC) columns, such as amide or zwitterionic stationary phases, are among the most suitable options for the separation of highly polar and charged analytes. In HILIC, retention increases with compound polarity, and the presence of multiple phosphate groups further enhances retention. These methods typically employ a high initial proportion of organic solvent—such as acetonitrile, methanol isopropanol, ketones, or mixture thereof—while an increase in the aqueous component increases elution strength. The aqueous phase commonly includes a volatile buffer, such as ammonium acetate or formate, to maintain pH control and promote ionization. However, while HILIC columns can be operated over a wide range of aqueous contents, effective analyte retention typically occurs within a narrower composition window, which can complicate method development and analyte separation. In addition, HILIC separations are sensitive to changes in solvent composition and gradient conditions, often requiring extended column equilibration times. Another limitation is the need to dissolve samples in a high proportion of organic solvents, mimicking the initial mobile phase injection conditions and therefore diluting analyte concentration. Despite these challenges, HILIC remains one of the most effective approaches for the separation of highly polar and charged compounds [39–41].

**Fig. 6:**
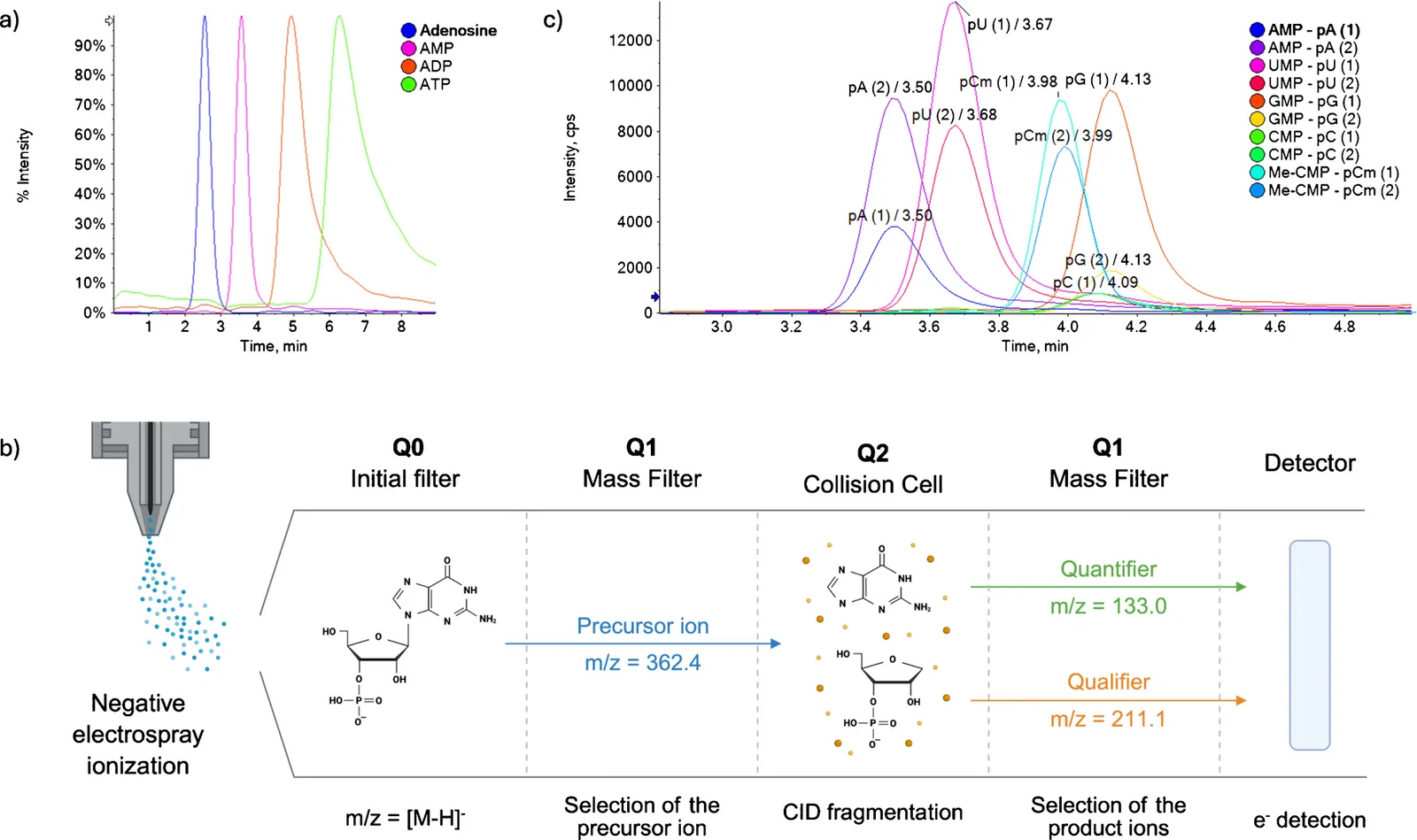
**a** Separation of adenosine-derived products (A, AMP, ADP, and ATP) using HILIC-MS in negative-mode ionization performed on a BEH amide column (Waters^®^) with an isocratic mobile phase of 100 mM ammonium formate in water (pH 3.5) and acetonitrile (40:60, v/v) at 0.2 mL/min and 35°C column temperature. **b** Overlay of extracted ion chromatogram (EIC) of nucleotide monophosphates including all the transitions acquired under the same HILIC-MS conditions. **c** Schematic representation of MRM transition development for nucleotides

In Fig. 6a, an overlay of the different adenosine nucleotides species is shown. As observed, increasing polarity and net negative charge result in stronger retention on this stationary phase and broader peaks. In contrast, analytes lacking phosphate groups are better separated by reversed-phase chromatography, particularly with stationary phases design to improve retention of polar analytes, where factors such as surface area and pore structure can also play a significant role [42].

#### MS detection

In this workflow, the analytes of interest belong to a predefined list of known compounds, allowing the use of certified reference standards for each species. The analysis is therefore considered a target analysis. For this reason, triple quadrupole mass spectrometers operated in multiple reaction monitoring (MRM) mode, coupled with electrospray ionization (ESI), are typically employed. This configuration enables the optimization of precursor-to-product ion transitions for each compound, maximizing detection sensitivity and selectivity. As a result, very low detection limits can be achieved with high analytical confidence, which is characteristic of target MS methods.

Analytes containing phosphate groups generally exhibit improved ionization efficiency in negative ion mode, which also enhances selectivity due to the reduced number of compounds ionizing under these conditions. In contrast, nucleosides and nucleobases can be analyzed in either positive or negative ionization mode depending on the other analyzed compounds [43].

This analytical approach also enables the detection and quantification of capping reagents, which can be optimized to assess their presence in the final product. Moreover, their digestion products can be monitored, as these species are expected to appear in the nucleotide pool following total digestion. Using certified reference standards, the multiple reaction monitoring (MRM) method can be systematically optimized for each nucleotide species. This process involves the selection of the most intense and chemically representative precursor ion, followed by the identification of the most abundant and structurally informative product ions. Typically, one transition is designated for quantification, whereas a second, independent transition is used for confirmation, as illustrated in Fig. 6c. The relative intensity ratio between quantifier and qualifier transitions is monitored as an additional identity criterion, ensuring high selectivity and reliable detection in complex matrices. This targeted MS/MS approach enables robust compound confirmation while maintaining excellent sensitivity and reproducibility [44]. Optimized MRM transitions are summarized in Supplementary Table S2, including specific acquisition parameters.

While this workflow provides high sensitivity and robust confirmation of nucleotide composition, it does not preserve any sequence-specific information. Consequently, it is primarily suited for identity confirmation and relative quantification of nucleotide modifications [45].

#### Case study: quantification of nucleotide modifications within the mRNA

This case study focuses on the quantification of nucleotide modifications incorporated into the mRNA structure, particularly for evaluating the efficiency of modification incorporation under different in vitro transcription (IVT) conditions, such as varying reagent concentrations. Following total enzymatic digestion of the mRNA into mononucleotides or nucleosides, the modified species of interest are included as targeted analytes within the analytical method. As illustrated in Fig. 6b as an example, 5-methylcytidine was quantified as a representative modified nucleotide, with the corresponding transitions integrated into the MRM detection method [44].

These modifications are typically incorporated at measurable levels across the mRNA sequence, depending on the transcript design. As a result, low limits of detection and quantification are generally unnecessary for their measurement, enabling their analysis using less complex mass spectrometer platforms, such as single-quadrupole instruments.

When appropriate chromatographic resolution is obtained, these nucleotide species can also be monitored using UV detection at 260 nm, providing an orthogonal cost-effective and robust strategy for the routine assessment of modification incorporation.

#### Case study: process-related impurities from IVT synthesis (residual rNTPs)

This case study examines the quantification of residual ribonucleotide triphosphates (rNTPs) originating from the IVT reaction, to demonstrate their effective removal from the final mRNA product [46]. Regulatory guidelines typically require such process-related impurities to be controlled below predefined thresholds, for example, below 0.1% of total impurities (generally expressed as g total impurities per g API × 100), assuming no significant toxicological concern. Meeting these specifications necessitates markedly lower limits of detection and quantification than those required for the analysis of major components.

A key analytical challenge arises from the presence of the intact mRNA transcript, which constitutes the predominant component of the sample matrix and may hinder the detection of low-level rNTPs. To mitigate such matrix effects and enable the use of small-pore chromatographic columns, a total enzymatic digestion strategy can be implemented. This approach converts the mRNA into smaller, non-interfering species, thereby enabling robust chromatographic separation and sensitive detection of residual triphosphates. It is essential to verify that nuclease treatment does not lead to degradation of the rNTPs themselves, ensuring the accuracy and reliability of the quantitative assessment.

### Workflow II: partial digestion and oligonucleotide mapping

#### Sample preparation to obtain oligonucleotides and analyte characteristics

Given the large size of therapeutic mRNA molecules, typically ranging from 1000 to 2000 nucleotides, direct sequence-level characterization of intact transcripts remains analytically challenging. Partial enzymatic digestion provides a middle-up strategy, in which long mRNA strands are cleaved into oligonucleotides of manageable length while preserving sequence context and positional information for downstream mapping and identification [47, 48].

This workflow leverages controlled partial digestion with sequence-specific ribonucleases (RNases). By selectively cleaving the mRNA at defined nucleotide motifs, these enzymes generate a pool of oligonucleotide fragments with peptide-like dimensions, while preserving sufficiently long sequence stretches to maintain meaningful positional information. Depending on the specificity of the RNase employed, both the number and length distribution of the resulting oligonucleotides can be modulated, directly impacting sequence coverage and mapping resolution. A detailed understanding of RNase cleavage preferences, reaction kinetics, and the nature of the digested products, including potential intermediate species, is essential for accurately predicting and interpreting the resulting oligonucleotide pool. To enhance enzyme accessibility and ensure uniform digestion, the mRNA is commonly denatured prior to enzymatic treatment, typically by heating to 90–95°C, to disrupt secondary and tertiary structures.

RNases can be broadly classified according to their increasing degree of sequence specificity. The enzyme with the lowest specificity is RNase A, which cleaves after pyrimidines (both cytidines and uridines) and generates fragments with a 3′-phosphate terminus [49]. More nucleotide-specific enzymes include RNase T1 (cleaving after guanosines) [50], RNase U2 (after adenosines) [51], cusativin (after cytidines) [52], and RNase MC1 (cleaving at uridines) [53, 54]. This last one generates a 5′-phosphate at the subsequent nucleotide different that the previous ones that leave a 3′-phosphate terminus. It is also noted that cusativin and MC1 may also present secondary cleavages depending on sequence context and experimental conditions. Higher sequence selectivity can be achieved with RNases that recognize dinucleotide or trinucleotide motifs. For example, RNase 4 cleaves between uridine–purine sequences (with primarily cleavage between U-A and secondary cleavage between U-G) [55], while colicin E5 cleaves between guanosine–uridine motifs, both generating a 3′-phosphate at the first nucleotide, and one of the most selective [56], MazF targets A-C-A motifs, leaving a 3′-phosphate terminus at the previous nucleotide [57]. Several RNases generate transient 2′3′-cyclic phosphate intermediates prior to formation of linear 3′-phosphate termini. Therefore, digestion conditions are typically optimized to drive reactions to completion, minimizing partially cleaved species and enabling confident oligonucleotide identification during downstream analysis. Table 2 summarizes the ribonucleases, detailing their cleavage specificities and typical digestion parameters, including enzyme-to-substrate ratio, incubation times, and buffer compositions. Most enzymes are compatible with Tris-HCl buffers (between 10 and 50 mM) supplemented with moderate ionic strength (typically 50–100 mM NaCl), while optimal pH conditions vary depending on the RNase and are adjusted accordingly to ensure efficient and controlled cleavages.

**Table 2:** Ribonucleases cleavage specificities, indicating the cleavage site (↓) and the position of the phosphate group (p), together with typical fragment ratios, digestion times, and buffer conditions

Enzyme	Cleavage	Typical ratios*	Digestion time (min)	Buffer (pH)
RNase A	-Cp ↓ and -Up ↓	1:100–10^3^	5–30	7.0–8.0
RNase T1	-Gp ↓	1:50–500	10–60	7.5
RNase U2	-Ap ↓	1:20–200	15–60	4.5–5.5
Cusativin	-Cp ↓	1:50–500	15–60	7.5
RNase MC1	-U ↓ p-	1:50–300	15–60	7.0–7.5
RNase 4	-Up ↓ A- (1°), -Up ↓ G- (2°)	1:20–200	30–90	7.5
Colicin E5	-Gp ↓ U-	1:10–100	30–120	7.5 **
MazF	-Np ↓ ApCpA-	1:20–200	30–120	7.5

* Enzyme-to-RNA ratios (w/w) should be optimized depending on transcript length and structure

** Addition of MgCl_2_ (1–5 mM)

#### LC methods

Ion-pair reversed-phase (IP-RP) liquid chromatography remains the most widely applied chromatographic approach for the separation of oligonucleotides [58–61]. This technique combines a reversed-phase stationary phase, typically C_18_, with the addition of an ion-pairing reagent to the mobile phase, which facilitates the interaction between the negatively charged phosphate backbone of oligonucleotides and the hydrophobic stationary phase. Among the ion-pairing reagents commonly used, alkylamines are preferred due to their volatility and, consequently, their compatibility with mass spectrometry (MS) detection. Triethylamine (TEA) is the most extensively used reagent [62]; however, alternatives such as diisopropylamine (DIPEA), dimethylbutylamine (DMBA), hexylamine (HA), and octylamine (OA) have also been reported [63]. The latter two, due to the limited volatility, are incompatible with MS detection and are therefore typically employed in UV-based methods, with acetate acting as the counterion buffer. The hydrophobic character of the alkyl substituents directly influences retention behavior, allowing fine control of oligonucleotide retention, particularly for longer sequences.

The retention mechanism in IP-RP chromatography is governed by a dynamic equilibrium involving the negatively charged oligonucleotide, the organic cation derived from the ion-pairing reagent present in the mobile phase, and the stationary phase. Increasing the hydrophobicity of the ion-pair agent enhances its adsorption onto the stationary phase, thereby increasing the effective cationic surface density and consequently strengthening oligonucleotide retention. However, this enhancement in retention is accompanied by reduced compatibility with MS detection and increased ion suppression effects. Typical alkylamine concentrations range from 5 to 15 mM (0.1–0.2% v/v) [64, 65]. A major disadvantage associated with the use of alkylamine-based ion-pairing reagents is their toxicity and their tendency to persistently contaminate chromatographic and MS systems, often requiring dedicated instrumentation or extensive cleaning protocols to ensure reproducible results.

Hexafluoroisopropanol (HFIP) is frequently employed as a mobile-phase modifier in IP-RP methods [66, 67]. Although HFIP does not directly contribute to analyte retention, it plays a crucial role in pH control, suppression of metal adduct formation, and enhancement of desolvation efficiency in the electrospray ionization source. These effects collectively lead to improved signal intensity and spectral quality. Typical HFIP concentrations range from 50 to 300 mM (0.5–3% v/v). However, the use of HFIP presents several drawbacks, including its relatively high cost, toxicity, and environmental concerns associated with fluorinated compounds, which has motivated ongoing efforts to identify more sustainable alternatives.

Chromatographic columns are specifically engineered for oligonucleotide analysis and incorporate bioinert surface treatments to minimize nonspecific adsorption and oxidative degradation. In addition, they feature enlarged pore size (typically around 300 Å) to facilitate the diffusion and effective retention of larger oligonucleotide species. In workflows involving enzymatic digestion, these larger pore dimensions also accommodate residual proteins, such as ribonucleases, that remain in the injected sample. This reduces steric exclusion effects and minimizes unintended interactions that could otherwise compromise chromatographic performance and reproducibility.

As an alternative approach, HILIC columns with similar enlarged pore sizes (around 300 Å) have been adapted for oligonucleotide separations [68]. The main advantage of HILIC lies in the use of cleaner mobile phases that do not rely on ion-pairing reagents, thereby minimizing LC–MS system contamination by ion-pairing reagents that may suppress ionization in positive ionization mode. However, HILIC neither inherently improves MS sensitivity, compared to IP-RP, nor does it reduce metal adduct formation, in fact, metal adducts can be more pronounced depending on the stationary-phase chemistry and elution conditions [69]. The applicability of HILIC for oligonucleotides strongly depends on the type of stationary phase. Amide and diol-based HILIC phases have been shown to enable the analysis of longer oligonucleotides, including RNA species approaching 100 nucleotides [70], whereas zwitterionic phases often exhibit anion-exchange activity and may require high salt concentrations to efficiently elute highly charged oligonucleotides. In addition, HILIC-based separations generally offer limited flexibility in gradient design compared to IP-RP chromatography. Consequently, while HILIC is well suited for specific applications such as short oligonucleotides or digest-based RNA mapping workflows, IP-RP chromatography remains the more versatile approach for comprehensive oligonucleotide mapping [71].

#### MS detection

For oligonucleotide analysis, mass spectrometry provides accurate molecular mass determination together with fragmentation information for sequence confirmation. For this reason, high-resolution mass spectrometry platforms capable of untargeted analysis are typically employed, allowing the simultaneous acquisition of MS and MS/MS spectra. High-resolution MS instruments operated at resolving power of 20,000 or higher are typically required. Quadrupole time-of-flight (QTOF) and Orbitrap-based instruments are the most commonly used technologies for this purpose, as they offer the mass accuracy and resolving power required to discriminate closely related oligonucleotide species [72, 73].

Soft ionization techniques, such as electrospray ionization (ESI), are preferred for oligonucleotide analysis. Due to their highly polar nature and the presence of multiple phosphate groups, oligonucleotides exhibit a strong tendency for deprotonation and are therefore most commonly analyzed in negative ionization mode, which also provides additional selectivity [74]. Compared to conventional untargeted metabolomics workflows, the mass acquisition range is typically extended, commonly spanning from *m*/*z* 300 up to 2000–2500, depending on the instrument configuration. Although intact oligonucleotides often exceed molecular weights of 2500 Da, they can still be detected within this range due to their ability to form multiply charged ions. The charge state can be determined from the isotopic cluster spacing, which corresponds to 1/*z*, allowing assignment of the charge state (*z*). The requirement for multiple charging imposes an upper limit on the size of oligonucleotides that can be effectively detected. Under negative ionization conditions, the theoretical maximum charge state is governed by the number of phosphate groups present in the molecule. Consequently, the maximum observable charge state (*z*_max_) is commonly on the order of the number of nucleotides minus 1 and can be slightly higher for enzymatically digested oligonucleotides carrying an additional terminal phosphate group. In practical terms, the accumulation of charges leads to increasing electrostatic repulsion, which further limits the attainable charge states [75].

Once the multiply charged ions are acquired in the MS spectra, deconvolution algorithms can be applied to reconstruct the neutral mass of the analyte. These observed *m*/*z* values follow the relationship *m*/*z* = (*M* − *z*·*H*)/*z*, where *M* is the molecular mass of the oligonucleotide, and *H* is the mass of a proton. The deconvolution algorithm assigns charge states (*z*) to the detected peaks and mathematically reconstructs the original molecular mass by collapsing these charge–state distribution into a zero-charge spectrum, thereby providing mass accuracy and simplifying interpretation. Sodium adducts, and to a lesser extent potassium adducts, are frequently observed. These species originate from the formation of stable oligonucleotide–Na⁺ or K⁺ complexes that persist through the ionization process. Although these adducts are already present in the raw spectra and tend to be more pronounced at lower charge states (higher *m*/*z*), they become more clearly resolved after deconvolution.

For MS/MS acquisition, two main strategies are commonly employed: data-dependent acquisition (DDA) and data-independent acquisition (DIA). In DDA, MS/MS spectra are acquired for selected precursor ions, typically the most intense ones, resulting in to relatively clean fragmentation spectra that can be directly associated with individual precursors. This facilitates straightforward oligonucleotide mapping using established scoring algorithms, although low-abundance species may be missed. In contrast, DIA fragments all ions within predefined *m*/*z* windows across the full mass range, providing more comprehensive coverage but generating highly complex MS/MS spectra composed of fragments from multiple coeluting and multiply charged precursors [76, 77]. Consequently, data analysis requires advanced computational strategies to infer precursor–fragment relationship, often based on chromatographic coelution, fragment ion correlation, or spectral libraries/predicted fragmentation patterns. These challenges are particularly pronounced for oligonucleotides due to their broad charge state distributions and partially overlapping fragmentation series. Several dedicated software solutions have therefore been developed to support oligonucleotide mapping workflows. Commercial platforms such as BioPharma Finder (Thermo Fisher Scientific) provide pipelines primarily optimized for DDA-based analysis, whereas SCIEX’s Biologics Explorer has recently expanded to support both DDA- and DIA-type acquisitions. Within the Waters ecosystem, waters_connect applications (including UNIFI, CONFIRM sequence, and MAP sequence) integrate accurate mass measurements with DIA (MS^E^)-based fragmentation data. Open-source tools such as the NucleicAcidSearchEngine (NASE from OpenMS) enable sequence identification from MS/MS data but remain predominantly optimized for DDA datasets, with DIA data analysis remaining an area of ongoing development [78, 79].

Regarding fragment ion assignment, the collision-induced dissociation (CID) fragmentation behavior of oligonucleotides was experimentally characterized by McLuckey et al. [80] and remains the most widely adopted framework for their interpretation. Backbone cleavage can occur at three different positions, generating fragment ions originating from the 5′ end (a, b, and c ions) and from the 3′ end (x, y, and z ions). In addition, base loss from a-type ions (denoted as a–B ions) is frequently observed. Fragment ions are annotated according to the corresponding nucleotide position within the oligonucleotide sequence, and neutral losses, such as water or phosphate groups, are also commonly detected. These fragments patterns are different for RNA compared to DNA due to the presence of the 2′-hydroxyl group. Similar to precursor ions, fragment ions may be multiply charged if they contain multiple phosphate groups. Conversely, fragments that remain singly charged while exhibiting high *m*/*z* values may fall outside the effective detection range of the mass spectrometer, thereby limiting fragment ion coverage and, ultimately, oligonucleotide sequence confirmation. Higher-charge-state precursor ions generally promote more extensive fragmentation, while certain nucleotide modifications (e.g., 2′-fluoro substitutions) may reduce fragmentation efficiency and complicate spectral interpretation.

**Fig. 7:**
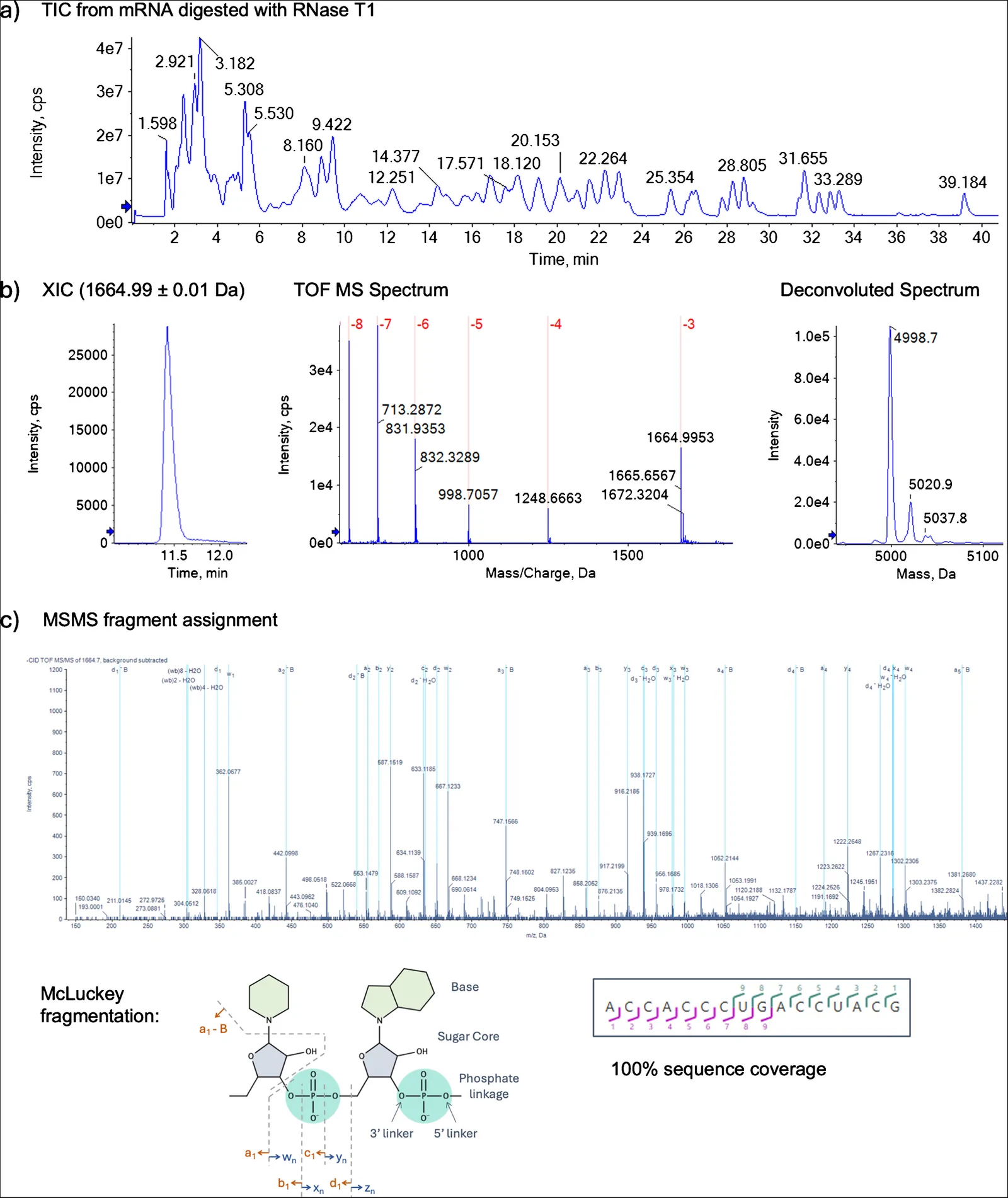
LC–MS/MS analysis of oligonucleotides generated by RNase T1 digestion of a 996-nt mRNA. **a** Total ion chromatogram (TIC) profile obtained by IP-RPLC using TEA/HFIP as ion-pairing system. **b** Extracted ion chromatogram (EIC), the corresponding mass spectrum, and deconvoluted mass spectrum of a 16-nt oligonucleotide. **c** MS/MS spectra with fragment assignments according to the McLuckey nomenclature, enabling sequence confirmation of the 16-nt oligonucleotide

The complete oligonucleotide characterization workflow is illustrated in Fig. 7, from the total ion chromatogram (TIC), MS spectra, deconvoluted neutral mass profiles, and MS/MS spectra used for fragment-based sequence confirmation to obtain the oligonucleotide’s sequence coverage.

Finally, many LC–MS systems used for oligonucleotide analysis incorporate an online UV detector prior to MS. UV absorbance at 260 nm is commonly used to monitor the chromatographic signal of nucleic acids, including oligonucleotides, providing an orthogonal and complementary readout that supports MS-based data interpretation, but does not in itself confirm molecular identity.

#### Case study: oligomapping

RNA sequence mapping presents additional challenges compared to peptide mapping. While proteins are composed of 20 amino acids, RNA sequences rely on only four nucleotides, resulting in a significantly higher probability of repeated motifs and reduced sequence uniqueness. As a consequence, achieving comprehensive sequence coverage is inherently more complex, and digestion strategies must be carefully designed to maximize the identification of informative oligonucleotides.

In this context, sequence coverage refers to the proportion of the transcript that can be confidently reconstructed from experimentally identified oligonucleotides. Unlike peptide mapping, a single enzymatic digestion is typically insufficient to achieve high coverage across the entire RNA sequence. Therefore, parallel digestion strategies using multiple ribonucleases are commonly employed to generate complementary oligonucleotide populations that collectively improve transcript coverage. These combined digests enable reconstruction of the full confirmed RNA sequences, or at least the most relevant regions such as the coding sequence and the UTR regions [47, 81].

A key concept in RNA mapping is the identification of unique oligonucleotides, defined as fragments that can be unambiguously assigned to a single position within the sequence. Very short oligonucleotides (typically < 4 nt) are generally excluded from analysis, as they lack sufficient sequence specificity and often occur at multiple positions within the transcript. In addition, isobaric oligonucleotides, distinct sequences sharing identical nucleotide composition and molecular weight, represent an analytical challenge, as they cannot be distinguished at the MS1 level and require confident MS/MS-based sequence confirmation.

Additional complexity arises from ionization efficiency and fragmentation behavior. As oligonucleotide length increases (typically beyond 20–30 nt, corresponding to 8–10 kDa), MS/MS-based sequence confirmation becomes increasingly difficult due to complex fragmentation patterns, reduced fragment ion coverage, and the presence of multiple charge states. In parallel, higher-molecular-weight oligonucleotides exhibit lower ionization efficiency and broader charge state distributions, which limit their detectability within the accessible *m*/*z* range. Together, these factors collectively define a practical upper mass limit for reliable detection and sequence confirmation under standard IP-RP-LC–MS conditions.

The relationship between RNase specificity, sequence coverage, and oligonucleotide properties is illustrated in Table 3, using a representative 996-nt mRNA example (nucleotide composition: 30.1% A, 12.7% U, 26.6% G, and 30.6% C). Different ribonucleases generate distinct digestion patterns, producing oligonucleotides with varying size distributions and degrees of uniqueness [47, 48]. Table 3 summarizes the number of generated oligonucleotides, their sizes and mass distributions, and the resulting sequence coverage based on both total and detectable fragments. Clear differences are observed depending on the RNase used. Enzymes such as RNase A, RNase T1, RNase U2, and cusativin generate numerous relatively short oligonucleotides, but only a limited fraction are unique, resulting in lower sequence coverage. In contrast, RNases with more specific cleavage patterns, such as RNase MC1 and RNase 4, produce fewer fragments but a higher proportion of unique oligonucleotides, leading to substantially improved theoretical coverage. However, enzymes that generate longer oligonucleotides (e.g., colicin E5 and MazF) exhibit a marked reduction in effective coverage under experimental conditions, as a significant portion of their digestion products falls outside the MS-accessible mass range. This comparison highlights that optimal sequence coverage is determined not simply by the number of fragments generated, but by a balance between fragment size, sequence uniqueness, and analytical detectability.

**Table 3:** Oligonucleotide characteristics and sequence coverage from theoretical digestion of a 996-nt mRNA using different RNases, with coverage reflecting unique and only detectable oligonucleotides under the actual LC–MS conditions

Enzyme	Total oligos	Length range (nt)	MW range (kDa)	Unique oligos	Theoretical sequence coverage	Detectable oligos	Restricted sequence coverage
RNase A	430	4–60	1.3–3.0	23	21%	22	15%
RNase T1	264	3–61	1.0–5.0	62	55%	61	49%
RNase U2	301	2–21	0.6–6.7	63	48%	63	48%
Cusativin	306	4–60	1.3–19.7	51	40%	50	34%
RNase MC1	127	3–62	0.9–20.3	74	86%	73	80%
RNase 4	70	2–62	0.6–20.3	60	96%	59	90%
Colicin E5	40	2–70	0.6–22.8	38	99%	34	73%
MazF	19	6–160	1.9–51.9	17	99%	9	21%

A more illustrative comparison between theoretical sequence coverage based on unique and detectable oligonucleotides amongst the 996-nt sequence is provided in Supplementary Figure S1.

By combining complementary RNases, the sequence coverage can be significantly improved by generating orthogonal oligonucleotide populations. In this dataset, combinations involving RNase 4 consistently yield the highest coverage reaching up to 92.6% (e.g., RNase 4-colicin E5 and RNase 4-cusativin), indicating that this enzyme plays a dominant role in generating informative oligonucleotide populations.

Notably, complete sequence coverage is rarely achieved, even when multiple RNases are combined, due to poorly cleaved or repetitive regions such as the 3′ poly(A) tail. These results indicate that an optimized combination of two RNases is generally sufficient to balance sequence coverage, analytical complexity, and experimental effort. Ultimately, the selection of RNases should be guided by the transcript regions of interest, such as the coding region, untranslated regions (UTRs), or terminal elements relevant for mRNA identity.

Given the complexity of oligonucleotide mixtures generated by enzymatic digestion, advanced data analysis strategies are required, as outlined in the previous section. Reliable sequence assignment depends on different parameter settings, including high mass accuracy (typically within 5–10 ppm for both precursor and fragment ions), appropriate handling of multiple charge states, and confident MS/MS fragmentation assignment. Statistical validation approaches such as false discovery rate (FDR) control, typically between 1 and 5%, can be implemented to ensure robust oligonucleotide identification. Additional experimental insights into charge state distribution and chromatographic behavior are provided in Supplementary Figure S2, supporting the observed correlation between oligonucleotide molecular weight and retention time.

In this context, literature reports consistently demonstrate that sequence coverage in LC–MS-based oligonucleotide mapping is highly dependent on the digestion strategy employed. While single RNase digestion generally provides limited coverage, the use of complementary RNases under optimized conditions enables substantially improved sequence coverage, often exceeding 70–90%. Such approaches have been successfully applied to a broad range of RNA molecules, including structured RNAs, tRNAs, and full-length mRNAs of up to approximately 3000 nt [82, 83].

Collectively, these criteria ensure robust, reproducible, and regulatory-aligned interpretation of mRNA mapping data derived from complex oligonucleotide mixtures by integrating confirmed oligonucleotide sequences to provide total transcript coverage.

#### Case study: magnetic beads-oligonucleotide capture for target mRNA region isolation

In addition to global mRNA mapping approaches, magnetic bead-based capture strategies can be employed to reduce sample complexity in oligonucleotide mapping experiments by selectively isolating specific regions of interest within the mRNA transcript, such as the 5′ cap or the 3′ poly(A) tail. These approaches are highly customizable, as the capture oligonucleotides must be specifically designed for each mRNA transcript to ensure efficient hybridization and selective enrichment [84, 85].

Biotinylated capture oligonucleotides are designed to hybridize selectively to the specific region of the target mRNA. Following hybridization, streptavidin-coated magnetic beads are used to enable selective immobilization of the target oligonucleotide–RNA duplex. Application of a magnetic field allows rapid separation of the bead-bound material from the supernatant, effectively removing the non-target oligonucleotides and substantially reducing sample complexity. After multiple washing steps to minimize nonspecific interactions, the captured oligonucleotide is eluted under mild denaturing conditions and subsequently analyzed by LC–MS [86].

Recovery yields are strongly dependent on the target region and the design of the capture oligonucleotide. For 3′ poly(A) tail isolation, standardized oligo(dT) designs are used, promoting an efficient hybridization, with recoveries between 60% and 90% [87]. In contrast, isolation of the 5′ cap-proximal region generally results in lower recoveries, between 40% and 70%, reflecting the increased structural constraints and design complexity associated with this region [88]. Despite these limitations, capture-based enrichment strategies significantly enhance the analytical characterization of specific mRNA regions compared with the analysis of unfractionated oligonucleotide pools. By reducing the background complexity, these protocols improve detection sensitivity and increase confidence in structural assignments. Further evaluation is required to determine whether such capture-based workflows are suitable for robust quantitative measurements or are better positioned for qualitative or semi-quantitative structural characterization.

### Workflow 3: intact mRNA analysis

#### Sample preparation

The third workflow focuses on the analysis of intact mRNA molecules, without prior enzymatic digestion, enabling assessment of the transcript as a complete entity. Although intact analysis does not provide sequence-level information, it serves as a valuable first-line quality control tool. Sample handling generally requires gentle conditions to prevent degradation, with mRNA maintained in RNase-free environments and appropriate buffering solutions. The absence of fragmentation preserves the native molecular integrity of the transcript, allowing evaluation of its overall structure, length, and homogeneity.

#### LC methods

For intact mRNA, liquid chromatography methods must be specifically adapted to retain very large, highly polar, and negatively charged molecules.

Three chromatographic strategies have been developed to assess transcript length, sample homogeneity, and degradation, and to support quantitative analysis of intact RNA. First, ion-pair reversed-phase (IP-RP) chromatography can be applied analogously to oligonucleotide analysis using stationary phases with large pore sizes and hydrophobic chemistries, including polymeric phases such as divinylbenzene or conventional C18 materials. These approaches enable the retention of high-molecular-weight RNA constructs while preserving the ion-pairing mechanism [89]. Second, size-exclusion chromatography (SEC) separates analytes exclusively based on their hydrodynamic size, minimizing interactions with the stationary phase and promoting gentle elution. Larger RNA molecules elute earlier due to size exclusion from the porous stationary phase, while smaller species penetrate the pores and elute later on [90, 91]. Third, anion-exchange chromatography (AEX) exploits the negatively charged phosphate backbone of RNA to retain transcripts on positively charged stationary phases. Elution is achieved by increasing ionic strength or adjusting pH to weaken electrostatic interactions [92]. Despite strong retention, separation selectively for intact transcripts of similar length remains limited.

Across all LC-based approaches, the use of bio-inert column hardware is essential, and pre-conditioning with concentrated samples is often required to saturate residual active sites and minimize nonspecific interactions, thereby improving injection-to-injection reproducibility. This is particularly critical for RNA analysis, where nonspecific adsorption (NSA) to metal surfaces has been widely reported and can lead to analyte loss, peak tailing, and reduced sensitivity. Multiple recent studies have elucidated the mechanisms underlying NSA and proposed effective mitigation strategies, including the use of hybrid organic–inorganic surface technologies and bio-inert system components [93–95].

A common limitation of IP-RP, SEC, and AEX is their restricted resolving power for intact RNA constructs with small differences in length. In practice, LC-based separation is typically limited to transcripts that differ substantially in size, reflecting the limited selectivity of these techniques for very large RNA constructs.

#### Alternative CE methods

Capillary electrophoresis (CE) offers substantially higher resolution for intact RNA transcripts and represents the only analytical technique capable of adequately resolving single-stranded RNA in the approximate range of 1000 to 10,000 nt. CE separations rely on electrophoretic mobility, which is governed by the charge-to-size ratio and molecular conformation of the analyte. Capillary zone electrophoresis (CZE) employs bare silica capillaries and can be operated under native or denaturing conditions, separating analytes on combined size and charge effects. Capillary gel electrophoresis (CGE) introduces a polymeric sieving matrix the enables size-dependent separation, analogous to slab-gel electrophoresis but in an automated capillary format.

Compared to LC, CE-based methods provide superior resolution, faster analysis time, and require only minimal sample volumes (nanoliters). In addition, CE can often be applied directly to complex matrices without extensive sample cleanup.

Despite these advantages, CE also presents important practical limitations. The high viscosity of polymer matrices used in CGE can negatively impact method robustness, capillary lifetime, and ease of operation. Detection sensitivity and dynamic range are often constrained by the available detection modes, and reliable quantitative performance may be challenging. In addition, CE methods require specialized instrumentation and expertise, and the lack of broadly accepted calibration standards for long RNA molecules complicates absolute sizing and interlaboratory comparability [96–98]. These factors currently limit the widespread routine application of CE for intact mRNA analysis, despite its superior resolving power.

#### Detection method

UV detection at 260 nm is commonly used for intact mRNA analysis, leveraging the intrinsic absorbance of nucleic acids [99]. Fluorescent labeling strategies, including the use of intercalating dyes or 3′/5′-end fluorophores, can substantially enhance analytical sensitivity, lower limits of detection, and enable accurate quantification at the nanogram level. In addition, fluorescent detection facilitates multiplexed analyses, as multiple transcripts can be labeled and detected simultaneously [100].

Mass spectrometry is generally not applicable to intact mRNA due to several intrinsic limitations, predominantly the extremely high molecular weight of full-length transcripts (>10,000 Da), which requires extensive multiple charging of the phosphate backbone for ionization under negative ionization conditions. This requirement results in highly heterogeneous charge state distributions and, combined with inefficient desolvation during electrospray ionization, severely compromises MS detectability and spectral interpretability. Although recent instrumental advances, including improvements in ion transmission and extended *m*/*z* detection ranges, have expanded the analysis of high-mass biomolecules, ionization efficiency remains the primary limiting factor for intact high-molecular-weight RNA analysis [101, 102].

Consequently, transcript length determination or molecular weight analysis based on chromatographic retention times or electrophoretic mobility is performed relative to RNA ladder standards. Calibration curves correlating retention or migration behavior with nucleotide length are generated and compared with those of the individual mRNAs.

In this context, alternative detection approaches such as multi-angle light scattering (MALS), typically coupled to LC, can be employed for the characterization of intact RNA molecules. MALS enables the direct determination of absolute molecular weight in solution based on light scattering intensity, without relying on ionization or calibration standards. However, MALS does not provide structural information and requires well-resolved samples, as co-elution or aggregation can affect the accuracy of molecular weight determination [103–105].

#### Case study: integrity of final transcript evaluation

**Fig. 8:**
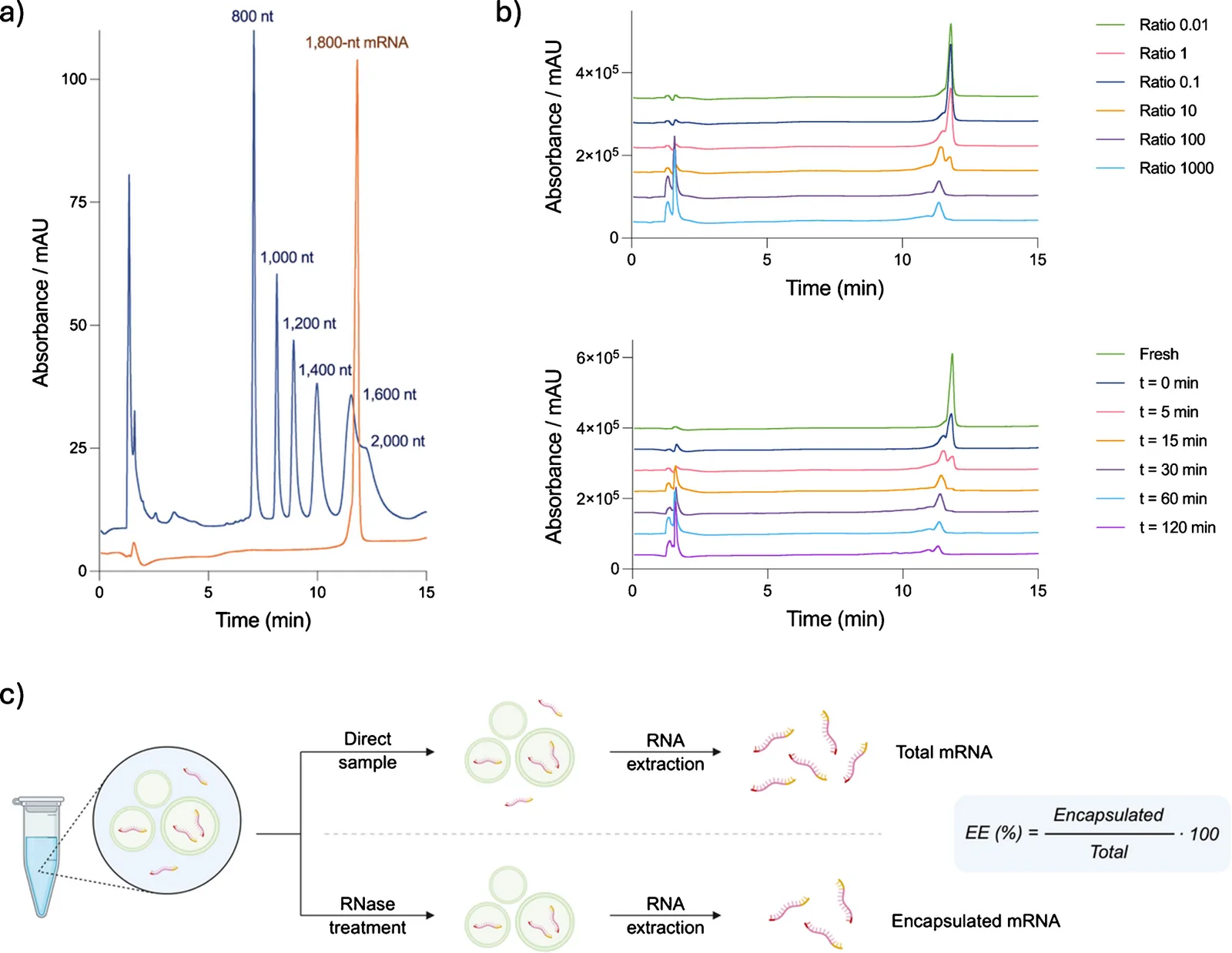
**a** IP-RPLC analysis of intact 1800-nt mRNA together with an RNA ladder performed on a DNAPac RP column using 200 mM TEAA aqueous buffer (adjusted in pH 7) and acetonitrile as mobile-phase components, with UV detection at 260 nm. **b** Digestion studies showing the overlay chromatograms of different ratios studies and digestion times of an 1,800-nt mRNA digested with RNase T1 at 37°C. **c** Theoretical scheme for encapsulation efficiency calculation with RNase treatment and nanoparticle disruption for RNA extraction

Assessment of intact mRNA integrity represents a fundamental component in quality control workflows. This analysis not only confirms the presence of the full-length transcript but also enables detection of potential truncations or degradation products that may compromise biological efficacy. Separation techniques, including IP-RP, SEC, or AEX chromatography, allow simultaneous visualization of the main transcript and minor fragments, enabling the detection of subtle degradation events. UV detection at 260 nm provides rapid and orthogonal confirmation of nucleic acid content. As illustrated in Fig. 8a, IP-RPLC analysis of an 1800-nt mRNA together with an RNA ladder enables clear identification of the intact transcript and assessment of its size and integrity.

In addition, comparison against calibrated RNA ladders enables accurate estimation of transcript length, a critical parameter for regulatory compliance. Beyond basic verification, intact transcript analysis supports storage stability studies, evaluation of buffer and formulation effects, and detection of aggregation or conformational heterogeneity. Although, this workflow does not deliver sequence-level information, it ensures that only high-quality, homogeneous mRNA is advanced to downstream processes, such as enzymatic mapping or encapsulation.

#### Case study: monitoring digestion for bottom-up studies

Intact mRNA analysis plays a pivotal role in optimizing partial digestion strategies. By monitoring the full-length transcript, analysts can verify that RNase treatment is digesting properly, which is essential for generating reproducible oligonucleotide pools suitable for sequence mapping. Key experimental parameters, including enzyme-to-substrate ratio, incubation time, and temperature are systematically monitored to balance fragment size with sequence specificity. The presence of intact transcripts in post-digestion samples indicates incomplete cleavage and serves as a diagnostic indicator for adjusting reaction conditions. As illustrated in Fig. 8b, IP-RPLC analysis of an 1800-nt mRNA after RNase T1 treatment shows disappearance of the intact transcript peak with increasing digestion ratios and times, while the resulting short oligonucleotide fragments (typically <100 nt) are not retained under these conditions and elute near the void volume as unresolved signals. Incorporation of intact mRNA assessment into the workflow therefore enhances the robustness and reproducibility of partial digestion LC–MS/MS analyses.

#### Case study: encapsulation efficiency strategy

Determination of mRNA encapsulation efficiency within nanoparticles, or other equivalent controlled delivery system, is critical for determining the therapeutic potential of the formulation. Intact mRNA analysis of both encapsulated and unencapsulated (free) mRNA enables evaluation of formulation efficiency and supports optimization of nanoparticle composition, component ratios, and loading conditions. In addition, intact mRNA analysis can reveal incomplete encapsulation, aggregation, or degradation events occurring during formulation.

Encapsulation efficiency is commonly determined by distinguishing free (non-associated) mRNA from the total mRNA content. This is most frequently achieved using indirect fluorescence-based assays, such as RiboGreen-based methods, which selectively report on accessible mRNA and are widely used for lipid nanoparticle (LNP) formulation [106, 107].

Alternatively, capillary electrophoresis (CE) enables direct quantitative analysis of free mRNA without prior extraction, as its charge-based separation allows reliable discrimination of intact transcripts even in complex nanoparticle matrices. In many cases, nanoparticle components do not significantly interfere with CE injection or detection, enabling direct assessment of unencapsulated mRNA [108].

For LC-based methods, nanoparticle components often interfere with chromatographic retention and detection, requiring the use of orthogonal strategies.

An alternative workflow combines selective RNase digestion with subsequent nanoparticle disruption, as illustrated in Figure 8c. In this approach, accessible, non-encapsulated mRNA is selectively degraded by RNase treatment, while transcripts protected within nanoparticles remain intact. Following RNase inactivation, nanoparticles are disrupted to release the encapsulated mRNA, which is then quantified. In parallel, total mRNA is determined from an equivalent sample that undergoes nanoparticle disruption without prior RNase treatment.

The nanoparticle disruption step must be carefully optimized to ensure efficient release of encapsulated mRNA while preserving transcript integrity. For lipid nanoparticles, mild surfactants or detergents are commonly employed for this purpose [109, 110]. In some workflows, RNase digestion is combined with organic extraction using chaotropic lysis reagents to isolate protected mRNA; however, such protocols may introduce matrix effects due to residual solvents in the injected samples.

To mitigate these effects, alternative extraction workflows, including spin-column-based or magnetic bead extraction methods, can be adapted to quantify encapsulated and free mRNA with reduced matrix interference. Accurate determination of encapsulation efficiency further supports downstream studies, such as in vitro translation and stability testing, by providing precise input for dose normalization. Moreover, intact transcript analysis facilitates comparative evaluation of different formulations, excipient systems, or storage conditions, thereby supporting reproducibility and regulatory compliance. When integrated with oligonucleotide mapping and nucleotide-level characterization, all provide a comprehensive assessment of both the molecular-level and formulation-level quality attributes of therapeutic mRNA.

## Linking analytical workflows to critical quality attributes and regulatory perspectives

The analytical workflows described throughout this work can be directly linked to the evaluation of specific critical quality attributes (CQAs) of mRNA-based drug products. Each workflow targets a distinct level of molecular information, spanning global transcript integrity, sequence confirmation, chemical modification content, and impurity profiling.

As summarized in Table 4, intact mRNA analysis primarily addresses CQAs related to transcript length, integrity, and degradation, whereas partial digestion and oligonucleotide mapping enable detailed assessment of sequence fidelity, modified nucleotide incorporation, and site-specific heterogeneity. The integration of multiple, complementary LC-based analytical approaches is therefore essential to ensure comprehensive CQA coverage across the mRNA life cycle.

**Table 4:** Mapping of the critical quality attributes (CQAs) to the corresponding analytical workflows

Quality	Attribute		Aim	Methodology	Workflow
Chemical identity	Sequence		To confirm that the nucleotide sequence corresponds exactly to the intended transcript with no unintended variants	High-throughput sequencing (HTS), Sanger sequencing, or reverse transcription polymerase chain reaction (RT-PCR) sequencing	
				IP-RP LC HRMS	II
	5′-end cap		To confirm presence, structure, and integrity of the 5′-cap	HILIC LC MS/MS	I
				IP-RP LC HRMS	II
	Nucleotide modifications		To verify the presence and identity of modified nucleotides	HILIC or RP-LC MS/MS	I
	3′-end poly(A) tail		To confirm poly(A) tail presence and length	IP-RP LC HRMS	II
				IP-RP, SEC, or AEX with UV	III
				CE UV/fluorescence	III
Assay	RNA content, integrity, transcript length, or encapsulation efficiency		To quantitatively determine RNA amount, assess structural integrity and size, and verify efficient encapsulation (if applicable)	UV spectrophotometry	-
				RT-qPCR or RT-dPCR	-
				Agarose gel electrophoresis	-
				CE-UV/FLU	III
				IP-RP, SEC, or AEX with UV	III
Impurities	Product-related	Double-stranded RNA	To monitor dsRNA that may increase immunogenicity or safety	Immunoblot or enzyme-linked immunosorbent assay (ELISA)	-
		Aggregates and % fragments	To evaluate RNA fragmentation and aggregation that may impact stability, potency, and safety	Agarose gel electrophoresis	-
				IP-RP, SEC, AEX, or CE with UV/FLU	III
	Process-related	Residual DNA	To ensure effective removal of template DNA to minimize risk	Quantitative PCR (qPCR)	-
		Non-incorporated nucleotides and capping agents	To control residual small-molecule reagents from transcription and capping steps that may affect safety or quality	HILIC or RP-LC MS/MS	I
		T7 RNA polymerase	To confirm removal of enzymes to prevent immunogenicity or safety concerns	ELISA	-
		Residual solvents	To ensure solvent levels meet official limits, safe for patient exposure	USP 467/Ph. Eur. 2.4.24	-
Potency	Protein expression		To demonstrate biological activity of the RNA through protein expression	Cell-based assay	-
Safety	Endotoxin		To verify that levels are below acceptable limits, ensuring patient safety	USP 85/Ph. Eur. 2.6.14	-
	Bioburden		To assess and control microbial contamination prior to sterilization	USP 61, 62, 1115/Ph. Eur. 2.6.12, 2.6.32	-
Other	pH		To confirm that it is within a range that ensures stability, compatibility, and safety	USP 791/Ph. Eur. 2.2.3	-
	Appearance		To verify visual quality attributes (clarity, color, particulate matter), indicating product integrity and suitability for use	USP 631, 788, 790/Ph. Eur. 2.2.1, 2.2.2, 2.9.19	-

Regulatory agencies, including the EMA and US Food and Drug Administration (FDA), emphasize the need for comprehensive characterization of mRNA drug substances and drug products, encompassing identity, purity, integrity, and manufacturing consistency. Particular emphasis is placed on control of the final product, as well as on the detection, identification, and quantification of impurities and degradation products. The application of orthogonal analytical methods is strongly encouraged to ensure robustness and confidence in quality assessments, especially for complex modalities such as mRNA therapeutics. Looking ahead, analytical strategies are expected to increasingly focus on workflows integration, higher levels of automation, and improved throughput to support both development and routine quality control. In parallel, continued harmonization of regulatory expectations will be critical to facilitate method standardization and ensure data comparability across laboratories. Ongoing advances in LC–MS instrumentation and data analysis capabilities are anticipated to further reinforce the central role of these techniques in mRNA characterization.

## Conclusions

This review underscores the necessity of integrated, multilevel analytical strategies to address the unique characterization challenges posed by therapeutic mRNA. Given their molecular complexity, structural heterogeneity, and sensitivity to degradation, no single analytical method is sufficient to comprehensively assess mRNA critical quality attributes (CQAs). Instead, complementary LC-based workflows provide an orthogonal framework that enables robust evaluation across molecular, structural, and formulation-related dimensions.

By combining nucleoside-level analysis, partial digestion with oligonucleotide mapping, and intact mRNA characterization, LC-based and LC–MS methodologies collectively support the assessment of identity, integrity, chemical modification content, and impurity profiles throughout the mRNA life cycle. The case studies presented herein illustrate how these workflows can be strategically aligned with specific CQAs, emphasizing a fit-for-purpose analytical philosophy rather than reliance on a single universal approach.

Looking ahead, future advances in mRNA analytics are expected to focus on greater workflow integration, automation, and standardization, alongside continued improvements in MS-compatible separations and data analysis tools. Such developments, together with increasing regulatory harmonization, will be essential to ensure method comparability and robust quality control as mRNA therapeutics continue to evolve in complexity and clinical scope.

## Supplementary Information

Below is the link to the electronic supplementary material.

Supplementary file1 (DOCX 670 KB)

## Data Availability

No new data were generated or analyzed in this study.
