# Supplementary material for: From nucleotides to intact transcripts through oligonucleotides: Integrated chromatographic strategies for therapeutic mRNA characterization
Source: Anal Bioanal Chem. 2026 Jun 23;418(18):5811–34. doi: 10.1007/s00216-026-06615-x (PMC13541854; doi:10.1007/s00216-026-06615-x)
Supplement: Supplementary file 1 [file 216_2026_6615_MOESM1_ESM.docx]

**Supplementary information**

**Title**

From nucleotides to intact transcripts through oligonucleotides: Integrated chromatographic strategies for therapeutic mRNA characterization

**Authors**

Mar Losada, Gemma Gotor, Salvador Borrós and Cristina Fornaguera*

*Grup d'Enginyeria de Materials (Gemat), Institut Químic de Sarrià (IQS), Universitat Ramon Llull (URL), Barcelona, Spain.*

**corresponding author: cristina.fornaguera@iqs.url.edu*

**Table S1.** Exact masses, LogP values, and chemical formulas of canonical nucleobases, nucleosides, and mono-, di- and trinucleotides, including selected modified species relevant to mRNA analysis.

| **Compound** | **Nucleobases** | | | **Nucleosides** | | |
| --- | --- | --- | --- | --- | --- | --- |
|  | **Formula** | **Exact mass** | **LogP** | **Formula** | **Exact mass** | **LogP** |
| A | C₅H₅N₅ | 135.0545 | −0.5 | C₁₀H₁₃N₅O₄ | 267.0968 | −1.5 |
| U | C₄H₄N₂O₂ | 112.0273 | −1.0 | C₉H₁₂N₂O₆ | 244.0695 | −1.8 |
| G | C₅H₅N₅O | 151.0494 | −0.7 | C₁₀H₁₃N₅O₅ | 283.0917 | −1.9 |
| C | C₄H₅N₃O | 111.0433 | −1.3 | C₉H₁₃N₃O₅ | 243.0855 | −2.0 |
| Ψ | C₄H₄N₂O₂ | 112.0273 | −1.0 | C₉H₁₂N₂O₆ | 244.0695 | −1.8 |
| m¹Ψ | C₅H₆N₂O₂ | 126.0429 | −0.6 | C₁₀H₁₄N₂O₆ | 258.0851 | −1.6 |
| m⁵C | C₅H₇N₃O | 125.0589 | −1.0 | C₁₀H₁₅N₃O₅ | 257.1011 | −1.8 |

| **Compound** | **Monophosphates** | | | **Diphosphates** | | |
| --- | --- | --- | --- | --- | --- | --- |
|  | **Formula** | **Exact mass** | **LogP** | **Formula** | **Exact mass** | **LogP** |
| A | C₁₀H₁₄N₅O₇P | 347.0631 | −3.1 | C₁₀H₁₅N₅O₁₀P₂ | 427.0294 | −4.3 |
| U | C₉H₁₃N₂O₉P | 324.0359 | −3.0 | C₉H₁₄N₂O₁₂P₂ | 404.0026 | −4.0 |
| G | C₁₀H₁₄N₅O₈P | 363.058 | −3.3 | C₁₀H₁₅N₅O₁₁P₂ | 443.0243 | −4.5 |
| C | C₉H₁₄N₃O₈P | 323.0519 | −3.2 | C₉H₁₅N₃O₁₁P₂ | 403.0186 | −4.1 |
| Ψ | C₉H₁₃N₂O₉P | 324.0359 | −3.0 | C₉H₁₄N₂O₁₂P₂ | 404.0026 | −4.0 |
| m¹Ψ | C₁₀H₁₅N₂O₉P | 338.0515 | −2.9 | C₁₀H₁₆N₂O₁₂P₂ | 418.0182 | −3.8 |
| m⁵C | C₁₀H₁₆N₃O₈P | 337.0675 | −3.1 | C₁₀H₁₇N₃O₁₁P₂ | 417.0342 | −4.0 |

| **Compound** | **Triphosphates** | | |
| --- | --- | --- | --- |
|  | **Formula** | **Exact mass** | **LogP** |
| A | C₁₀H₁₆N₅O₁₃P₃ | 506.9957 | −5.8 |
| U | C₉H₁₅N₂O₁₅P₃ | 483.9695 | −5.5 |
| G | C₁₀H₁₆N₅O₁₄P₃ | 522.9906 | −6.0 |
| C | C₉H₁₆N₃O₁₄P₃ | 482.9855 | −5.6 |
| Ψ | C₉H₁₅N₂O₁₅P₃ | 483.9695 | −5.5 |
| m¹Ψ | C₁₀H₁₇N₂O₁₅P₃ | 497.9851 | −5.3 |
| m⁵C | C₁₀H₁₈N₃O₁₄P₃ | 497.0011 | −5.5 |

**Table S2.** Multiple reaction monitoring (MRM) transitions of the canonical nucleosides and nucleotides (mono-, di-, and triphosphates), including precursor and product ions, assigned formula and collision energies. Transitions were optimized on a SCIEX 7600 triple quadrupole mass spectrometer with ion trap operated in negative ion mode.

| **Compound** | **Precursor ion** | **Product ion** | **Assigned Formula** | **CE (V)** |
| --- | --- | --- | --- | --- |
| A | 266.6 | 134.0 | C_5_H_4_N_5_^-^ | -28 |
|  |  | 107.0 | C_8_H_7_N_9_^-^ | -42 |
| U | 243.5 | 110.0 | C_4_H_2_N_2_O_2_^2-^ | -18 |
|  |  | 152.1 | C_6_H_4_N_2_O_3_ | -18 |
| G | 282.6 | 151.1 | C_5_H_5_N_5_O | -22 |
|  |  | 134.0 | C_5_H_3_N_5_ | -42 |
| C | 242.7 | 183.0 | C_7_H_9_N_3_O_3_^-^ | -20 |
|  |  | 110.1 | C_4_H_4_N_3_O^-^ | -16 |
| AMP | 346.4 | 211.1 | C_8_H_6_N_5_O_2_^-^ | -22 |
|  |  | 134.0 | C_5_H_4_N_5_^-^ | -40 |
| UMP | 323.3 | 211.1 | C_5_H_8_O_7_P^-^ | -20 |
|  |  | 111.0 | C_4_H_3_N_2_O_2_^-^ | -30 |
| GMP | 362.4 | 211.1 | C_5_H_8_O_7_P^-^ | -22 |
|  |  | 133.0 | C_5_H_2_N_4_O^-^ | -48 |
| CMP | 322.4 | 211.1 | C_5_H_8_O_7_P^-^ | -22 |
|  |  | 139.0 | C_8_H_6_N_5_O_2_^-^ | -30 |
| ADP | 426.3 | 408.0 | C_10_H_12_N_5_O_9_P_2_^-^ | -24 |
|  |  | 134.1 | C_5_H_4_N_5_^-^ | -28 |
| UDP | 403.5 | 305.0 | C_5_H_10_NO_10_P_2_^-^ | -20 |
|  |  | 111.0 | C_4_H_3_N_2_O_2_^-^ | -30 |
| GDP | 442.5 | 150.1 | C_5_H_4_N_5_O^-^ | -32 |
|  |  | 424.9 | C_10_H_12_N_5_O_10_P_2_^-^ | -30 |
| CDP | 402.5 | 305.0 | C_5_H_10_NO_10_P_2_^-^ | -24 |
|  |  | 110.0 | C_4_H_4_N_3_O^-^ | -28 |
| ATP | 506.3 | 408.1 | C_10_H_12_N_5_O_9_P_2_^-^ | -27 |
|  |  | 134.1 | C_5_H_4_N_5_- | -32 |
| UTP | 483.3 | 384.9 | C_9_H_11_N_2_O_11_P_2_ | -26 |
|  |  | 111.0 | C_4_H_3_N_2_O_2_- | -32 |
| GTP | 522.2 | 424.1 | C_11_H_14_N_4_O_10_P_2_^-^ | -26 |
|  |  | 150.24 | C_5_H_4_N_5_O^-^ | -34 |
| CTP | 482.3 | 384.66 | C_9_H_12_N_3_O_10_P_2_^-^ | -26 |
|  |  | 110.04 | C_4_H_4_N_3_O^-^ | -38 |

**Figure S1**. Theoretical illustration of sequence coverage as a function of RNase specificity.

(A) Sequence coverage calculated based on all unique oligonucleotides generated by each RNase, reflecting the theoretical maximum coverage achievable from cleavage specificity alone.

(B) Sequence coverage considering only unique oligonucleotides within the experimentally accessible LC–MS range, highlighting the impact of detectability constraints on effective coverage.

**Figure S2.** Figure S4. IP-RPLC analysis of RNase T1 digestion products.

(A) Distribution of oligonucleotide charge states as a function of retention time, showing the presence of multiple charge states across the chromatographic profile and their variation with elution behavior.

(B) Overall charge state distribution of detected oligonucleotides, highlighting the predominance of moderately charged species and the contribution of multicharged ions to signal detection.

(C) Correlation between molecular weight and retention time, demonstrating that higher molecular weight oligonucleotides elute at longer retention times due to stronger interactions with the ion-pairing system.
